# Lipophagy deficiency exacerbates ectopic lipid accumulation and tubular cells injury in diabetic nephropathy

**DOI:** 10.1038/s41419-021-04326-y

**Published:** 2021-10-30

**Authors:** Yachun Han, Shan Xiong, Hao Zhao, Shikun Yang, Ming Yang, Xuejing Zhu, Na Jiang, Xiaofen Xiong, Peng Gao, Ling Wei, Ying Xiao, Lin Sun

**Affiliations:** 1 Department of Nephrology, The Second Xiangya Hospital, Central South University, Key Laboratory of Kidney Disease and Blood Purification, Changsha, Hunan China; 2 Department of Nephrology, The Third Xiangya Hospital, Central South University, Changsha, Hunan China

**Keywords:** Chronic kidney disease, Diabetes complications

## Abstract

Autophagy-mediated lipotoxicity plays a critical role in the progression of diabetic nephropathy (DN), but the precise mechanism is not fully understood. Whether lipophagy, a selective type of autophagy participates in renal ectopic lipid deposition (ELD) and lipotoxicity in the kidney of DN is unknown. Here, decreased lipophagy, increased ELD and lipotoxcity were observed in tubular cells of patients with DN, which were accompanied with reduced expression of AdipoR1 and p-AMPK. Similar results were found in db/db mice, these changes were reversed by AdipoRon, an adiponectin receptor activator that promotes autophagy. Additionally, a significantly decreased level of lipophagy was observed in HK-2 cells, a human proximal tubular cell line treated with high glucose, which was consistent with increased lipid deposition, apoptosis and fibrosis, while were partially alleviated by AdipoRon. However, these effects were abolished by pretreatment with ULK1 inhibitor SBI-0206965, autophagy inhibitor chloroquine and enhanced by AMPK activator AICAR. These data suggested by the first time that autophagy-mediated lipophagy deficiency plays a critical role in the ELD and lipid-related renal injury of DN.

## Introduction

Diabetic nephropathy (DN) is a severe complication of diabetes mellitus and has become the most common primary disease leading to end-stage renal disease worldwide [1]. The importance of tubular injury in the progression of DN and the role of inflammation, mitochondrial oxidative stress and autophagy disorders in the pathogenesis of DN was confirmed in our previous studies [2, 3]. Emerging evidence suggests that the dyslipidemia in patients with DN was associated with estimated glomerular filtration rate (GFR), inflammation, fibrosis and disease progression [4, 5]. High-plasma triacylglycerols(TG) and non-esterified fatty acids (NEFAs) results in the lipid accumulation and lipotoxicity in non-adipose tissues [6, 7]. Moreover, renal cells were damaged by ectopic lipid deposition (ELD) associated with lipotoxicity *via* activating inflammation, reactive oxygen species (ROS) production, mitochondrial dysfunction and cell-death in kidney of DN [4, 8–12]. However, the precise mechanism through which lipid disorders impact the kidney damage in DN remains unclear.

Selective autophagy, lipophagy, plays a key role in regulating the synthesis and degradation of intracellular lipid droplets (LDs) in hepatocytes [13], macrophage foam cells [14], etc. Lipophagy refers to that when LDs are sequestered by autophagosomes, which fuse with lysosomes to form autolysosomes, TG and cholesteryl esters in LDs are then degraded by lysosomal hydrolases in autolysosomes into free fatty acids (FFAs) that are recycled back into the cytosol for mitochondrial β-oxidation [15, 16]. The activation of lipophagy was dependent on the environment and energy requirement of different types of cells [17], such as during prolonged starvation and the early phase of lipid challenge [16, 18]. Defective lipophagy were reported to be linked with liver steatosis, obesity, atherosclerosis and the metabolic syndrome of aging [14, 18–21]. Interestingly, increased lipophagy may relieve insulin resistance by decreasing lipid accumulation in adipocytes in patients with type 2 diabetes [22]. However, the level of lipophagy in kidney tissues in DN and whether it is involved in the kidney injury is unknown.

In this study, lipophagy deficiency was observed in the tubular cells of patients with DN and db/db mice, which was accompanied by significantly ELD, oxidative stress, apoptosis. These damages were ameliorated in tubular cells as the levels of lipophagy was increased by AdipoRon administration, while the effects were blocked partially by AdipoR1 siRNA, autophagy inhibitor, ULK1 inhibitor and enhanced by AMPK activator. These data demonstrated by first time that disrupted lipophagy modulated by AdipoR1/AMPK pathway plays a key role in ELD and lipotoxicity in tubular cells of DN.

## Results

### Renal injury and ELD were associated with lipophagy deficiency in patients with DN

**Fig. 1:**
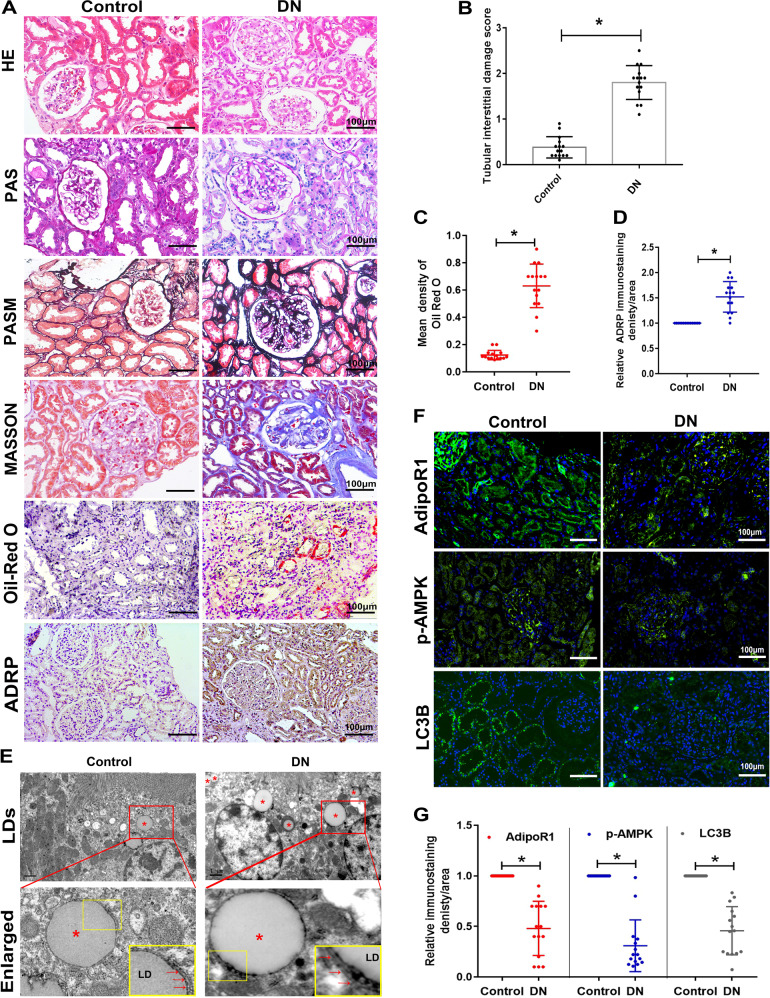
Decreased lipophagy and pathological changes in the kidney tissues of patients with DN. HE, PAS, PASM, Masson trichrome, Oil-red O staining and immunohistochemical analysis of ADRP in the renal biopsy tissues of patients with DN and control (magnification, ×200) (**A**). Tubular interstitial damage score (**B**). Quantification of Oil-Red O density (**C**) and ADRP expression (**D**). Ultrastructural analysis of lipid deposition in the kidney tissues of patients with DN and control (magnification, ×10,000) * indicates LDs, red arrows indicate double-membrane autophagic vacuoles containing lipids (**E**). IF analysis and semiquantification of AdipoR1, p-AMPK and LC3B in the kidney tissues of patients with DN and control individuals (**F**, **G**). Values are the mean ± SD; *p < 0.05 versus control. *n* = 15.

Elevated blood glucose, hemoglobin A1c(HbA1C), TG, total cholesterol (TC), low-density lipoprotein cholesterol (LDL-C) and 24-h urine protein and decreased albumin were observed in patients with DN (Table 1). Significant typical nodular glomerulosclerosis, basement membrane thickening, focal tubular atrophy, interstitial fibrosis, severe tubular injury and obvious lipid deposition occurred in the kidney of patients with DN (Fig. 1A–D). Positive correlations were observed in tubular interstitial damage and lipid deposition (*r* = 0.872), tubular interstitial damage and adipose differentiation related protein (ADRP) expression (*r* = 0.907), lipid deposition and ADRP expression (*r* = 0.807) in kidney tissues of patients. Transmission Electron Microscope (TEM) detection showed that some LDs were sequestrated by double-membrane organelles, namely, autophagosomes, in both groups. More LDs and less lipophagy were represented in the kidney of DN, which indicated that lipid deposition and insufficient lipophagy may be closely related to pathological injury in the kidney of DN (Fig. 1E).

**Table 1:** Clinical characteristics of the patients with DN and the patients with minor lesions who did not have diabetes.

	Control	DN	*p*
BMI (kg/m^2^)	23.1 ± 3.1	24.7 ± 4.2	0.25
Blood glucose (mmol/L)	4.68 ± 0.52	8.6 ± 0.23	<0.0001
HBA1c (%)	4.75 ± 0.46	8.2 ± 0.53	<0.0001
Total cholesterol (mmol/L)	6.31 ± 0.14	7.24 ± 0.19	<0.0001
Triglyceride (mmol/L)	2.65 ± 0.38	4.22 ± 1.6	0.0009
LDL (mmol/L)	2.1 ± 0.64	2.9 ± 0.88	0.0082
ALT (IU/L)	18.3 ± 4.3	17.8 ± 5.4	0.7811
AST (IU/L)	24.1 ± 8.7	23.8 ± 7.67	0.9209
Albumin (g/L)	39.1 ± 3.5	32.3 ± 1.9	<0.0001
BUN (mmol/L)	5.81 ± 0.41	6.03 ± 0.22	0.07777
Scr (μmol/L)	92.4 ± 8.9	95.17 ± 7.54	0.3656
UA (μmol/L)	346 ± 67	409 ± 83	0.0299
Urine protein (g/24 h)	3.14 ± 1.31	4.38 ± 1.09	0.0088
Red blood cells count in urine sediment(/mL)	15010 ± 998	11003 ± 1034	<0.0001
Systolic pressure (mmHg)	119.12 ± 4.5	148.19 ± 2.5	<0.0001
Diastolic pressure (mmHg)	82.14 ± 1.67	96.13 ± 2.07	<0.0001

Values are the mean ± SD.

*HBA1c* glycosylated hemoglobin, *LDL* low-density lipoprotein, *ALT* alanineaminotransferase, *AST* aspartate aminotransferase, *BUN* blood urea nitrogen, *Scr* serum creatinine, *UA* uric acid.

### The expression of AdipoR1, p-AMPK, and LC3B in the kidneys of patients with DN were noticeably decreased and the AdipoR1 expression was correlated with renal function as well as autophagosome assembly

**Fig. 2:**
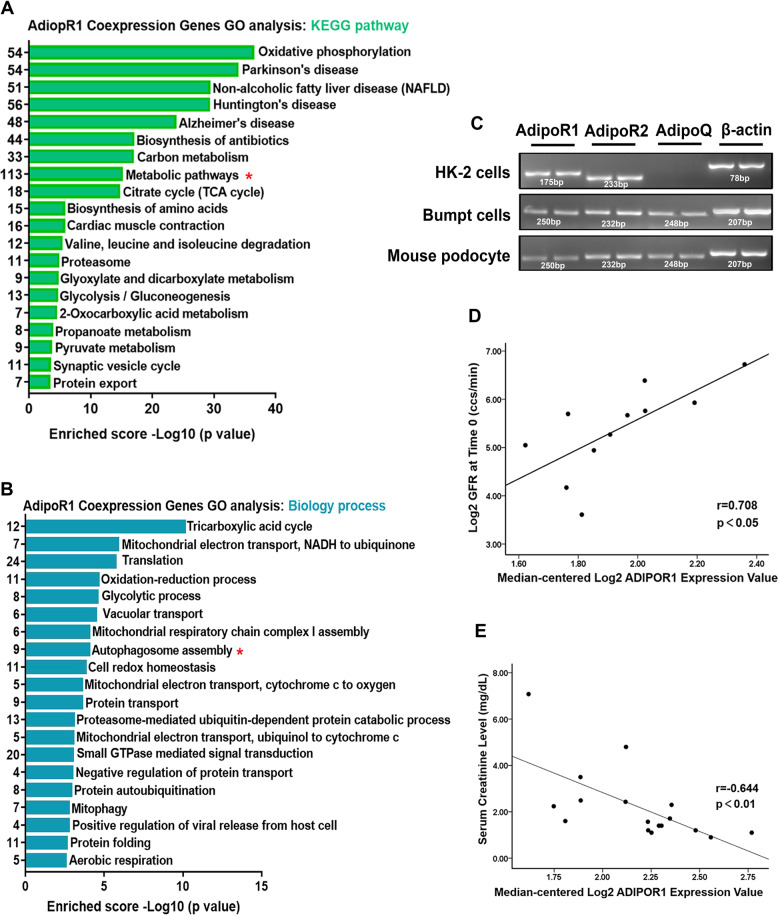
Analysis of *AdipoR1* genes. GO analysis of genes coexpressed with *AdipoR1* (positive correlation coefficient ≥0.6) according to KEGG pathway (**A**) and Biology process (**B**). Detection of *AdipoR1*, *AdipoR2* and *AdipoQ* by PCR amplification of DNA from HK-2 cells, BUMPT cells and mouse podocytes (**C**). Correlation of AdipoR1 expression and GFR (**D**) and serum creatinine level (**E**) in patients with DN and control individuals from the Nephroseq database. Values are the mean ± SD; *r*: correlation coefficient; *p < 0.05 versus control.

Moreover, considering that AdipoR1 is involved in the regulation of lipid metabolism and autophagy, AdipoR1 was detected to explore the mechanism of lipophagy deficiency in the DN kidney tissues. Immunofluorescence (IF) analysis revealed that AdiopR1 expression was reduced in kidneys of patients with DN (Fig. 1F, G). Additionally, AMPK is an important modulator of autophagy and a downstream regulator of AdipoR1, the level of its phosphorylation in the kidney and its relationship with lipid deposition and tubular injury were detected. The expression of p-AMPK and LC3B in the kidneys of patients with DN was noticeably decreased (Fig.1F, G). Here, AdipoR1 expression was negatively correlated with lipid deposition (*r* = −0.653), ADRP expression (*r* = −0.683), and positively correlated with p-AMPK expression (*r* = 0.849) and LC3B expression (*r* = 0.804). P-AMPK expression was negatively correlated with tubular interstitial damage (*r* = −0.868), lipid deposition (*r* = −0.774), ADRP expression (*r* = −0.863), but positively correlated with LC3B expression (*r* = 0.893). LC3B expression was negatively correlated with tubular interstitial damage (*r* = −0.761) and lipid deposition (*r* = −0.618). Meanwhile, Gene Ontology analysis was carried out, and the results showed that some of the genes positively coexpressed with *AdipoR1* (correlation coefficient ≥0.60) were enriched in metabolic pathway and autophagosome assembly according to Kyoto Encyclopedia of Genes and Genomes (KEGG) pathway (Fig. 2A) and biology process analysis (Fig. 2B), respectively. Additionally, real-time PCR detection determined the expression of *AdipoR1*, *AdipoR2* and *AdipoQ* in the HK-2 cells, BUMPT cells (a mouse proximal tubule-derived cell line) and mouse podocytes, *AdipoR1* and *AdipoR2* were expressed in all of these cells’ line, while *AdipoQ* was expressed in BUMPT cells and mouse podocytes (Fig. 2C). Moreover, according to the Nephroseq database, AdipiR1 expression was positively correlated with GFR (*r* = 0.708, Fig. 2D) and negatively correlated with serum creatinine (Scr) level (*r* = −0.644, Fig. 2E) in patients with DN, indicating that the expression of AdipoR1 was closely correlated with renal function in patients with DN.

### Increased lipophagy by AdipoRon ameliorates renal injury, lipid deposition, oxidative stress, and apoptosis in the kidney of db/db mice

Treatment of db/db mice with AdipoRon for 4 weeks, the body weight, kidney weight and Scr level of db/db mice showed no significant change from those of the untreated group. Blood glucose, HbA1C, TG and 24-h albuminuria were increased in db/db mice compared with control mice, but these changes were all reversed by AdipoRon treatment. Serum adiponectin levels decreased obviously in db/db mice compared with control mice, and there was no significant difference in adiponectin levels between the db/db mice treated with AdipoRon and the untreated db/db mice (Table 2).

**Table 2:** Characteristics of the different groups’ mice.

Group	db/m	db/db Cont	db/db+ AdipoRon	p*	*p*^#^
*n*	12	12	12		
Body weight (g)	29.8 ± 1.5	53.3 ± 5.2	51.5 ± 3.2	<0.0001	0.4531
Kidney weight (mg)	203 ± 5.8	204.7 ± 7.3	203 ± 9.2	0.8471	0.8471
Blood glucose (mmol/L)	8.5 ± 0.5	29.89 ± 3.6	21.7 ± 3.7	<0.0001	<0.0001
HbA1c (%)	4.1 ± 0.2	9.2 ± 0.4	8.6 ± 0.1	<0.0001	<0.0001
Triglyceride (mmol/L)	0.98 ± 0.2	1.96 ± 0.2	1.63 ± 0.3	<0.0001	0.0017
Total cholesterol (mmol/L)	1.43 ± 0.2	3.46 ± 0.3	3.2 ± 0.4	<0.0001	0.13
Scr (μmol/L))	16.6 ± 2.9	19.1 ± 3.6	17.8 ± 3.3	0.1641	0.6
24 h albuminuria (mg/day)	10.17 ± 1.7	222.8 ± 35.2	157.4 ± 20.7	<0.0001	<0.0001
Serum adiponectin (μg/mL)	10.9 ± 0.8	4.6 ± 0.4	4.7 ± 0.5	<0.0001	0.9156

Values are the mean ± SD.

p*: db/m versus db/db; *p*^#^: db/db versus db/db + AdipoRon.

*HBA1c* glycosylated hemoglobin, *Scr* serum creatinine.

**Fig. 3:**
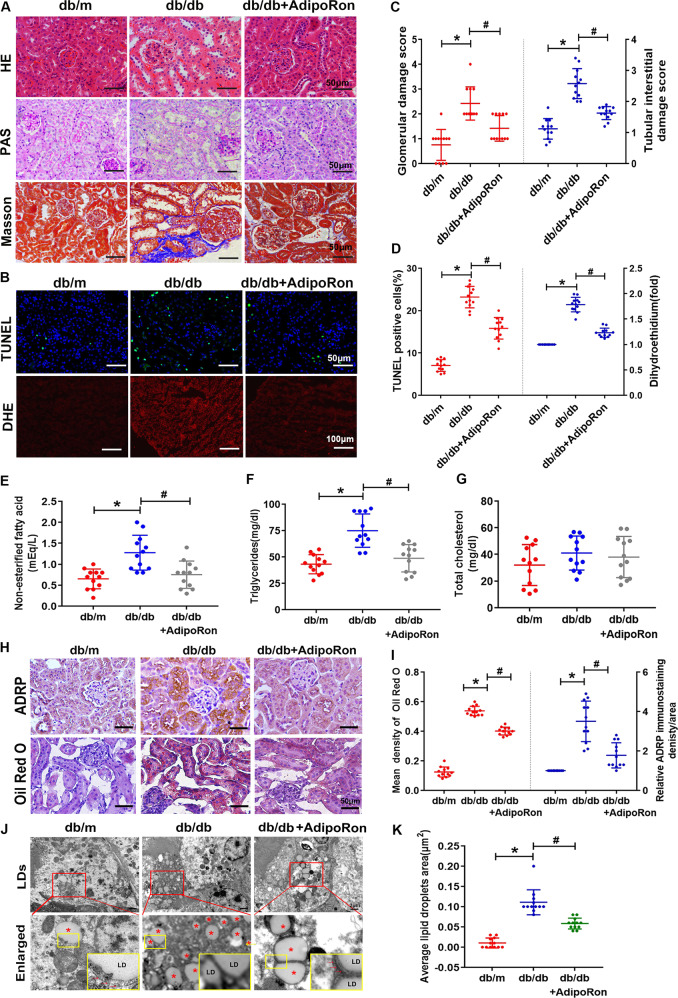
AdipoRon increases lipophagy, ameliorated lipid deposition and renal pathological injury of db/db mice. Kidney sections stained with HE, PAS, Masson trichrome, TUNEL (magnification, ×400) and DHE (magnification, ×200) (**A**, **B**). Glomerular damage score and tubular interstitial damage score (**C**). TUNEL-positive cells in the kidney tissues and quantitative analysis of intrarenal DHE (**D**). Renal non-esterified fatty acids in the kidney tissues (**E**). Renal triglycerides (**F**). Renal total cholesterol (**G**). IHC analysis of ADRP and Oil-Red O staining in the kidneys (**H**). Semiquantification of the mean density of Oil-Red O and ADRP expression in the kidney (**I**). TEM analysis of lipid deposition in the kidney tissues of three groups of mice (magnification, ×10,000). * indicates the LDs, and red arrows indicate double-membrane autophagic vacuoles containing lipidsnamely, lipophagy (**J**). Average LD area in the kidney tissues of the three mouse groups (**K**). Values are the mean ± SD; *p < 0.05 versus control group; #*p* < 0.05 versus db/db group. *n* = 12.

In addition, glomerular hypertrophy, mesangial cells and matrix significantly increased in some glomeruli and some dilated or atrophied tubules and renal interstitial fibrosis were noted in the kidneys of db/db mice, which were all partially alleviated following AdipoRon treatment (Fig. 3A, C). Additionally, cell apoptosis and oxidative stress in the kidneys were enhanced in db/db mice compared with control mice, while were attenuated by AdipoRon (Fig. 3B, D). Moreover, AdipoRon administration decreased intrarenal lipid concentrations, which were represented by the decreased renal NEFA and TG in db/db mice (Fig. 3E, F), while there was no significant alteration in TC (Fig. 3G). Further analysis revealed lipid deposition in the kidneys of db/db mice via IHC for ADRP detection and Oil-Red O staining. AdipoRon dramatically ameliorated this enhancement (Fig. 3H, I). Ultrastructural analysis revealed an increased number of LDs in the kidneys of db/db mice, and fewer LDs in the kidneys of db/db mice treated with AdipoRon. Importantly, in the kidneys of control mice and db/db mice treated with AdipoRon, clear autophagosomes or autolysosomes that engulfed LD were noted (Fig. 3J, K).

### AdipoRon upregulated the expression of p-AMPK, p-ULK1 and autophagosome-associated proteins, while reduced fibrosis in the kidney of db/db mice

**Fig. 4:**
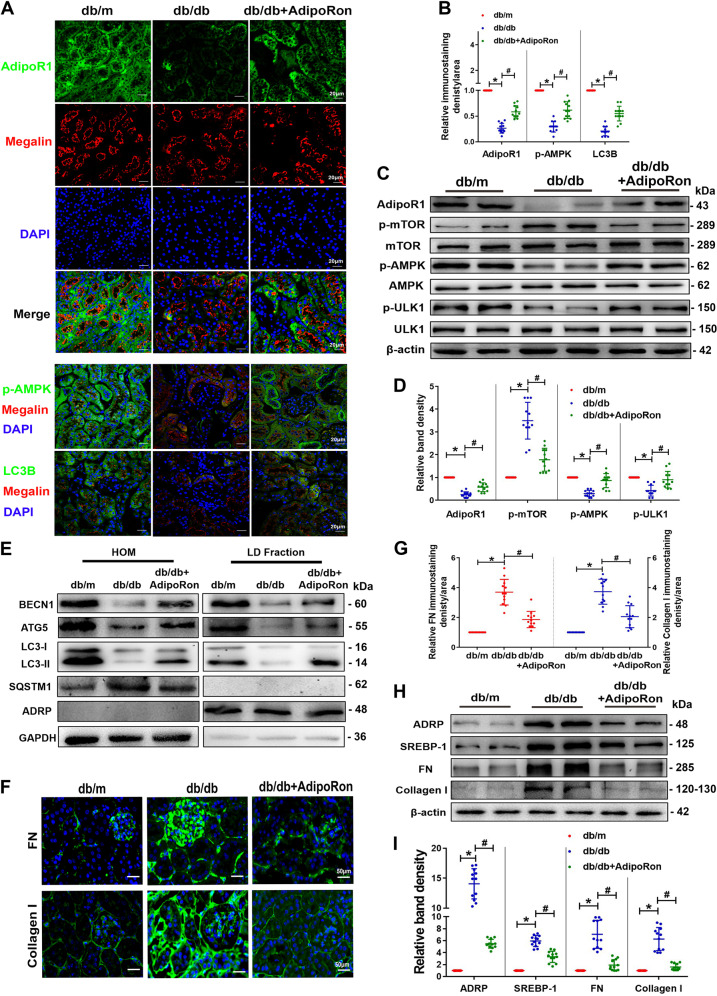
AdipoRon increased lipophagy, activated AdipoR1/AMPK pathway and alleviated fibrosis in the kidney of db/db mice. Representative IF images of AdipoR1, p-AMPK, and LC3B in the kidney tissues (magnification, ×400) (**A**). Semiquantification of the IF analysis of AdipoR1, p-AMPK and LC3B (**B**). WB analysis and quantification of AdipoR1, p-mTOR, p-AMPK and p-ULK1 expression in the kidneys (**C**, **D**). WB analysis of the indicated molecules using homogenates (HOM) and isolated LD fractions from the kidneys (**E**). Representative IF images and semiquantification of FN and Collagen I in the kidneys (**F**, **G**). WB analysis and quantification of the ADRP, SREBP-1, FN and Collagen I levels (**H**, **I**). Values are the mean ± SD; *p < 0.05 versus control, ^#^*p* < 0.05 versus db/db group. *n* = 12.

The decreased expression of AdipoR1, p-AMPK and LC3B and increased p-mTOR level in db/db mice were all partially reversed by AdipoRon treatment (Fig. 4A–D). Similar alterations were also showed in autophagosome-associated proteins such as beclin1 (BECN1), autophagy related 5 (ATG5) and LC3-II in the LD fraction. Sequestosome 1 (SQSTM1) was not detected. The purification degree of the LD fraction was determined by the abundant ADRP expression and the absence of GAPDH (Fig. 4E). Fibronectin (FN)and Collagen I were reduced by AdipoRon treatment (Fig. 4F, G). Protein alterations in ADRP, sterol regulatory element binding protein-1 (SREBP-1), FN and Collagen I were further confirmed by WB analysis (Fig. 4H, I).

### AdipoRon reversed the lipophagy alteration and lipid deposition in HK-2 cells exposed to high-glucose (HG) ambience

**Fig. 5:**
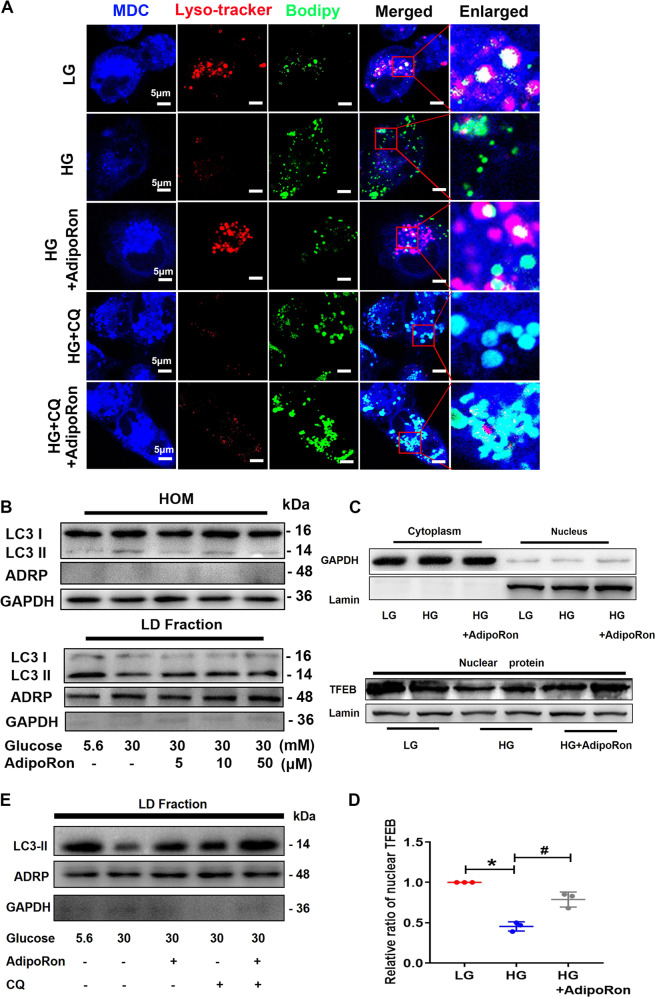
AdipoRon increased lipophagy in HK-2 cells exposed to HG environment. Representative images of intracellular autophagosome (MCD), lysosomes (Lyso-tracker) and lipid (Bodipy) co-localization in HK-2 cells in an HG environment with or without AdipoRon, CQ pretreatment (**A**). WB analysis of LC3II expression using HOM and isolated LD fractions from HK-2 cells exposed to HG conditions with or without different AdipoRon concentrations (**B**). WB and densitometric analysis of TFEB expression using nuclear protein from HK-2 cells exposed to HG conditions with or without AdipoRon (**C**, **D**). WB analysis of LC3II expression using isolated LD fractions from HK-2 cells exposed to HG conditions with or without AdipoRon and CQ pretreatment (**E**). Values are the mean ± SD; *p < 0.05 versus LG group, ^#^*p* < 0.05 versus HG group. *n* = 3.

**Fig. 6:**
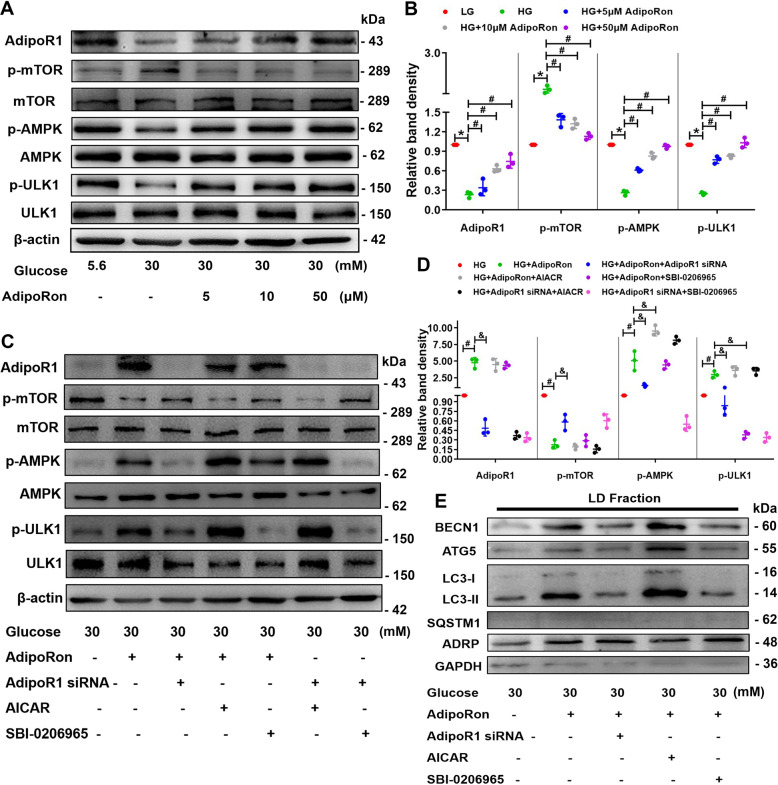
AdipoRon increased lipophagy through AdipoR1/AMPK pathway. WB and densitometric analysis of AdipoR1, p-mTOR, p-AMPK and p-ULK1 expression in HK-2 cells exposed to HG conditions with or without different AdipoRon concentrations (**A**, **B**). WB and densitometric analysis of AdipoR1, p-mTOR, p-AMPK and p-ULK1 expression in HK-2 cells exposed to an HG environment pretreated with AdipoRon, AdipoR1 siRNA, AICAR or SBI-0206965 (**C**, **D**). WB analysis of BECN1, ATG5, LC3 and SQSTM1 using isolated LD fractions from HK-2 cells exposed to HG conditions with or without AdipoRon, AdipoR1 siRNA, AICAR and SBI-0206965 (**E**). Values are the mean ± SD; *p < 0.05 versus LG group, ^#^*p* < 0.05 versus HG group, ^&^p < 0.05 versus HG + AdipoRon group. *n* = 3.

The co-localization studies of autophagosomes, lysosomes and LDs were performed to identify whether LDs are associated with lipophagy. AdipoRon treatment increased autophagosomes (stained by monodansylca- daverine, MDC) and lysosomes (stained by Lyso-tracker), while decreased the number of LDs (stained by Bodipy). Moreover, AdipoRon treated cells exhibited more co-localization of LDs with autophagosomes and lysosomes (increased white stained regions). Additionally, inhibition of lysosomal degradation of LDs that sequestered autophagosomes by chloroquine (CQ) enhanced co-localization of LDs with autophagosomes (Fig. 5A). The upregulation of LC3-II levels in isolated LD fraction from HK-2 cells exposed to HG conditions with AdipoRon and CQ treatment were confirmed by WB analysis (Fig. 5B, E). Similar results were also seen in the expression of transcription factor EB(TFEB) in nuclear protein extracted from HK-2 cells under HG condition pretreated with AdipoRon (Fig. 5C, D). Additionally, a decreased expression of AdipoR1, p-AMPK and p-ULK1 as well as an increasing level of p-mTOR in total cell protein from HK-2 cells exposed to HG were found, while were reversed when pretreated with AdipoRon (Fig. 6A, B).

### The effects of AdipoRon on increasing lipophagy, reducing ELD and fibrosis in HK-2 cells were partially blocked by AdipoR1 siRNA and ULK1 inhibitor, while enhanced by AMPK activator

Effects of AdipoRon were abolished by pretreatment with AdipoR1 siRNA or ULK1 inhibitor SBI-0206965 and reinforced by AMPK activator AICAR (Fig. 6C, D). Similar alterations in BECN1, ATG5 and LC3-II were also observed in the LD fraction isolated from HK-2 cells exposed to HG after pretreated with AdipoR1 siRNA, SBI-0206965 and AICAR (Fig. 6E).

**Fig. 7:**
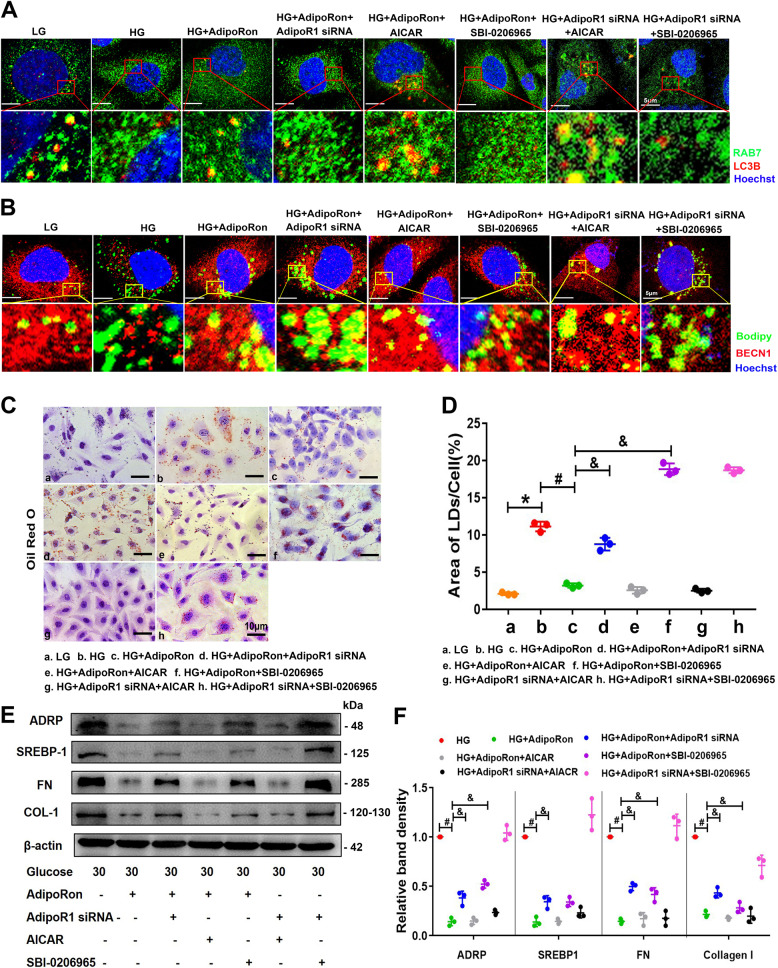
The effects of AdipoRon on increasing lipohagy and alleviating lipid deposition and fibrosis were partially blocked by ULK1 inhibitor, while enhance by AMPK activator. Confocal IF images showing the co-localization of RAB7 and LC3B and the co-localization of BECN1 and LDs (Bodipy) in HK-2 cells exposed to HG conditions and pretreated with or without AdipoRon, AdipoR1 siRNA, AICAR and SBI-0206965 (**A**, **B**). Representative images and semiquantification of the lipid deposition in HK-2 cells exposed to an HG environment pretreated with AdipoRon, AdipoR1 siRNA, AICAR and SBI-0206965 (**C**, **D**). WB and densitometric analysis of ADRP, SREBP-1, FN and Collagen I expressions in HK-2 cells exposed to HG conditions with or without AdipoRon, AdipoR1 siRNA, AICAR and SBI-0206965 (**E**, **F**). Values are the mean ± SD; *p < 0.05 versus LG group, ^#^*p* < 0.05 versus HG group. *n* = 3.

When intracellular LDs were engulfed by autophagosomes, ADRP was removed and dissociated from RAB7, the protein on the LD surface that subsequently interacts with autophagy proteins, such as SQSTM1/p62 and LC3-II [16]. The co-localization of RAB7 and LC3B, BECN1 and LDs (Bodipy stained) was decreased in HK-2 cells under HG conditions, and AdipoRon administration reversed these changes. However, these effects were partially abolished by pretreatment with AdipoR1 siRNA, SBI-0206965 and enhanced by AICAR (Fig. 7A, B). Similar alterations were also found in the intracellular lipid deposition (Fig. 7C, D), which was accompanied by the protein alterations of ADRP, SREBP-1, FN and Collagen I (Fig. 7E, F). Overall, AdipoRon activated AdipoR1/AMPK pathway to increase lipophagy that alleviated lipid deposition and fibrosis in HK-2 cells exposed to HG conditions pathway.

## Discussion

The present study demonstrated by first that the lipophagy deficiency in tubular cells was related to the ELD-associated kidney damage in both patients and mice with DN. Increasing the level of lipophagy by administration of AdipoRon through AdipoR1/AMPK pathway, which ameliorates lipotoxicity-associated tubular injury, oxidative stress and apoptosis. These data indicate that lipophagy dysfunction contributes to ELD-induced kidney injury in DN.

Lipophagy, a type of selective autophagy that sequesters LDs in autophagosomes for degradation by lysosomes [15]. It has been reported that lipophagy was associated with hepatic disorders, including liver steatosis, nonalcoholic fatty liver disease, and liver fibrosis [23, 24]. The methods of identifying lipophagy include Oil-Red O, BODIPY 493/503 or HCS LipidTOX staining, triglyceride content determination and TEM [15]. It also has been demonstrated that lipophagy is induced to breakdown LDs to maintain energy homeostasis under prolonged starvation in proximal tubule cells [15, 25]. Furthermore, lipophagy was inhibited under nutrient abundance since FFAs were not needed as an energy source for cells [26]. Chronic lipid exposure such as ELD might be a contributor to lipophagy disorder in type 2 diabetes [22]. Moreover, previous studies by our and other group have found that renal ELD and its associated lipotoxicity in the kidney are the main causes of the development of obesity-related nephropathy and type 2 DN [4, 27–29]. Abnormal autophagy was found to participate in kidney injury in DN in vivo and in vitro [25, 30, 31]. However, whether autophagy-mediated lipophagy involved in the pathogenesis and development of chronic kidney disease including DN still needs to be discussed. Here, we demonstrated that the reduced lipophagy and lipid deposition in the renal tissues of patients with DN were positively correlated with lipotoxicity induced tubular injury (Fig. 1).

On the other hand, decreased renal AdipoR1 expression was accompanied by increased renal TG levels in diabetic rats [32], while increasing AdipoR1 expression ameliorated lipotoxicity [33]. Here, we confirmed the expression of AdipoR1 in tubular cells, and Gene Ontology analysis showed that the genes positively correlated with AdipoR1 expression were enriched in metabolic pathways and autophagy formation, and the expression level of AdipoR1 was correlated with renal function in DN patients (Fig. 2). In addition, AdipoRon, an AdipoR1 activator, which can enhance glucose and lipid metabolism and inhibits lipotoxicity of diabetic mice through AdipoR1/ AMPK pathway [10, 34], it also might promote autophagy in renal epithelial cells [35]. However, it is not clear that whether enhanced autophagy-mediated lipophagy by AdipoRon might alleviate lipotoxicity induced renal injury in DN. In this study, we found that levels of lipophagy in kidney of diabetic mouse was increased by AdipoRon, which was accompanied by decrease in proteinuria and renal lipid contents levels as well as the alleviation of renal pathological injury, oxidative stress, apoptosis and fibrosis (Table 2 and Figs. 3 and 4). These data further demonstrated that pharmacological enhancement of the level of autophagy-mediated lipophagy could reverse the ELD and tubular cells injury in DN.

It is reported that AdipoR1 activation by AdipoRon could restore AMPK-mediated autophagosome formation and stimulate autophagosome clearance under diabetic conditions [36]. Additionally, the decreased AdipoR1 and AMPK activation led to a reduction in fatty acid oxidation and an increase in fatty acid synthesis, promoting the progression of type 2 diabetes [37, 38]. The increased AMPK phosphorylation and subsequently activated autophagy has been demonstrated to be a renal protection mechanism in DN [39, 40]. AMPK is a key enzyme that regulates autophagy, which is involved in the development of DN [41]. Furthermore, it is known that autophagy was promoted by AMPK through directly activating ULK1 phosphorylation, while inhibited by mTOR through disrupting the interaction between ULK1 and AMPK [42]. Additional, ULK1 plays an important role in regulating lipid metabolism in adipocytes by regulating autophagy [43]. Increased levels of p-AMPK and ULK1 were suggested to be related to the activation of lipophagy in hypothalamic neurons [44]. As expected, treatment of HK-2 cells with AdipoRon reversed HG-induced lipophagy dysfunction and increased lipid deposition, while these effects were partially blocked by pretreatment with autophagy inhibitor, but enhanced by AMPK activtor (Figs. 5–7). These data suggested that increased lipophagy by AdipoRon in tubular cells of DN might through activating AdipoR1/AMPK pathway.

Briefly, in this study we found that the lipophagy deficiency plays a critical role in the ELD and lipotoxicity in tubular cells of DN. AdipoRon can reduce intrarenal lipotoxicity-associated renal injury and fibrosis in DN through increasing lipophagy by activating AdipoR1/AMPK pathway. These findings suggest that targeting lipophagy might be a potential therapeutic strategy for DN and other ELD-related metabolic diseases.

## Materials and methods

### Antibodies and reagents

We used the following commercially available antibodies: The anti-AdipoR1 (ab126611), anti-FN (ab2413), anti-Collagen I (ab34710), anti-AMPKα1 + AMPKα2 (ab131512), anti-AMPK alpha 1 (phospho T183) + AMPK alpha 2 (phospho T172) (ab23875), anti-RAB7(ab50533) and anti-ADRP (ab52356) antibodies were from Abcam; the anti-phospho-AMPKα(Thr172) (2535), anti-mTOR(2972), anti-phospho-mTOR(Ser2448)(5536), anti-ULK1 (8054), anti-phospho-ULK1 (Ser555) (5869), anti-SQSTM1/p62(5114) and anti-LC3B (3868) antibodies were from Cell Signaling Technology; and the anti-SQSTM1/P62 (18420-1-AP), anti-LC3B (18725-1-AP), anti-Beclin1 (11306-1-AP), anti-ADRP (15294-1-AP), anti-ATG5 (10181-2-AP), anti-TFEB (13372-1-AP), anti-β-actin (60008-1-Ig), anti-GAPDH (60004-1-Ig) and anti-Lamin B(12987-1-AP) antibodies were from Proteintech. The anti-SREBP-1c(sc-13551) was from Santa Cruz Biotechnology. AdipoRon and CQ was obtained from Selleckchem. AICAR was provided by Apexbio Technology, SBI-0206965 was purchased from Sigma-Aldrich.

### Clinical data

Patients with DN were newly diagnosed by renal biopsy who used insulin to control blood glucose. Patients using lipid-lowering drugs, RAS inhibitors before biopsy and those with urinary tract infections, or with inflammatory, neoplastic, cardiovascular, hepatic, renal, lung or neuroendocrine diseases were excluded. The patients in the control group were the first diagnosed patients with primary glomerulonephritis of varying degrees of proteinuria and the pathological diagnosis was minimal change nephropathy. These patients were not treated prior to pathological diagnosis. The DN group included 15 patients (8 males and 7 females), the average age is 43.69 ± 7.62 years (34–57 years) and the control group included 15 patients (8 males and 7 females), the average age is 41.54 ± 7.66 years (32–56 years). The clinical experimental procedures were approved by the Ethics Committee of Second Xiangya Hospital, Central South University, and informed consent was obtained from all the patients.

### Animal experimental design

Fourteen-week-old male C57BLKS/J db/db and C57BLKS/J db/m mice were purchased from the Aier Matt Experimental Animal Company (China). They were randomly divided into the following three groups: a db/m group (control, *n* = 12), a db/db group receiving intragastric injection with AdipoRon (*n* = 12) and a db/db group that receive intragastric injection with 0.5% sodium carboxymethyl cellulose solution, which served as vehicle control (*n* = 12). AdipoRon (30 mg/kg) was dissolved in 0.5% sodium carboxymethyl cellulose solution and then provided to db/db mice once daily via oral gavage from 16 weeks of age for 4 weeks as described previously [45]. The mice were euthanized at 20 weeks of age. All animal procedures were approved by The Animal Care and Use Committee of Second Xiangya Hospital of Central South University.

### Assessment of physiological features

Body weights and blood glucose levels were measured twice per week. Twenty-four-hour urine samples were collected using metabolic cages before the mice were euthanized. Urine albumin concentrations were tested using an Albuwell M kit, and levels of serum creatinine were measured using HPLC as previous study [45]. The intrarenal lipid levels, namely FFAs, TG and TC using commercial kits (Wako, Osaka, Japan) according to manufacturer’s instructions.

### Morphological analysis

Renal tissues were fixed in 4% paraformaldehyde (PFA) and then embedded in paraffin. Four-micrometer thick paraffin-embedded tissue sections were prepared and subjected to hematoxylin-eosin (HE), periodic acid-Schiff (PAS), periodic acid-silver methe-namine (PASM) and Masson trichrome staining as previously described [2]. Tubulo-interstitial lesion and glomerular injury indices were evaluated as described previously [46, 47].

### IHC of renal tissue

Four-micrometer -thick paraffin-embedded renal tissue sections from human or mice were prepared for IHC studies, as previously described [3]. The sections were deparaffined in xylene, rehydrated in ethanol and soaked in 10 mM citrate buffer at 65 °C for antigen retrieval. After blocking endogenous peroxidase activity with 3% H_2_O_2_, the sections were exposed to 5% bovine serum albumin (BSA) and sequentially incubated with antibodies against ADRP (1:100, Proteintech, 15294-1-AP) overnight at 4 °C. The sections were then incubated with secondary antibodies conjugated with peroxidase, treated with diaminobenzidine, and finally counterstained with hematoxylin. Images were obtained using a Nikon microscope and analyzed by ImageJ.

### IF, apoptosis assay, and ROS accumulation determination of renal tissue

We performed IF analysis of AdipoR1(1:100, Abcam, ab126611), p-AMPK (1:200, Abcam, ab23875), LC3B (1:100, Proteintech, 18725-1-AP), FN (1:200, Abcam, ab2413), and Collagen I (1:200, Abcam, ab34710) by using 4% PFA-fixed, paraffin-embedded renal tissue sections (4 μm thick). Apoptotic renal cells were detected using the TUNEL assay with an In-Situ Cell Death Detection Kit (Roche Applied Science, China), in accordance with the manufacturer’s instructions. ROS in renal tissues were determined by oxidative fluorescent dye dihydroethidine (DHE, 1 μM, Invitrogen).

### WB analysis

RIPA buffer (CWBIO, China) supplemented with protease inhibitors and phosphatase inhibitors (CWBIO, China) was used to extracted protein from kidney tissues or HK-2 cells. Protein concentration was quantified by the BCA method. Equal amounts of protein were then analyzed by sodium dodecyl sulphate–polyacrylamide gel electrophoresis. Primary antibodies specific for AdipoR1 (1:3000, Abcam, ab126611), phospho-AMPKα(Thr172) (1:1000, Cell Signaling Technology, 2535), AMPKα1 + AMPKα2 (1:2000, Abcam, ab131512), phospho-ULK1 (Ser555) (1:1000, Cell Signaling Technology, 5869), ULK1 (1:1000, Cell Signaling Technology, 8054), ADRP (1:500, Abcam, ab52356), FN (1:1000, Abcam, ab2413), Collagen I (1:1000, Abcam, ab34710), LC3B (1:1000, Cell Signaling Technology, 3868), Beclin1 (1:800, Proteintech, 11306-1-AP), ATG5 (1:1000, Proteintech, 10181-2-AP), SQSTM1/ P62 (1:1000, Proteintech, 18420-1-AP, Cell Signaling Technology, 1:1000, 5114) and TFEB (1:1000, Proteintech, 13372-1-AP) were used. The bands were evaluated using a Tanon 5200 Multi instrument (Tanon Instruments, China). The band densities of target proteins were compared with those of β-actin (1:5000, Proteintech, 60008-1-Ig), GAPDH (1:5000, Proteintech, 60004-1-Ig) or Lamin B (1:1000, Proteintech, 66095-1-Ig) by using densitometry software (ImageJ).

### Cell culture and treatments

HK-2 cells were cultured as previously described [2]. Briefly, HK-2 cells were exposed to different concentrations of d-glucose (5.6 mM or 30 mM), with or without AdipoRon (5 nM, 10 nM, and 50 nM), AICAR (0.5 mM), SBI-0206965 (10 μm) or CQ (50 μm). For gene disruption, HK-2 cells were pretransfected with AdipoR1 siRNA using Lipofectamine 3000 (Invitrogen, USA) in accordance with the manufacturer’s protocol.

### Lipophagy analysis

Lipophagy was monitored with the following methods. (1) *Oil-Red O staining:* Five-micrometer-thick, 4% PFA-fixed, frozen renal tissue sections or 4% PFA-fixed HK-2 cells were rinsed with 60% isopropanol for 20–30 seconds, and stained with Oil-Red O dye solution for 15 min as previously described [12]. (2) *TEM*: Kidney samples were fixed in 2.5% glutaraldehyde in 0.1 M phosphate buffer (pH 7.4) overnight at 4 °C and fixed with 1% osmium tetroxide solution for 1 h. The samples were then dehydrated in ethanol and embedded in Epon 812. Ultrathin sections (~80 nm) were prepared and stained with uranyl acetate and lead citrate, followed by observation of the LD-associated double-membrane structure using TEM. (3) *WB analysis*: Lipid droplet components were isolated from mouse kidney samples and HK-2 cells as previously described [48]. Briefly, kidney samples from 8 kidneys were homogenized by ultrasonic disruption after being kept in icy buffer A (20 mM tricine, 250 mM sucrose, PH = 7.8) plus 0.2 mM phenyl methane sulfonyl fluoride (PMSF) for 20 min. Then, the samples were centrifuged at 100 × *g* for 10 min at 4 °C, and the supernatant was disrupted under high pressure. The homogenate was centrifuged at 3000 × *g* for 10 min at 4 °C to obtain the postnuclear supernatant (PNS) fraction. Ten milliliters of PNS and two milliliters of buffer B (20 mM HEPES, 100 mM KCl and 2 mM MgCl2, pH = 7.4) were placed into a SW40 tube and centrifuged by an ultracentrifuge at 100,000 × *g* for 60 min at 4 °C. The crude LD fraction was carefully collected from the top to the Eppendorf tube. Finally, the crude LD fraction was resuspended in 200 μL of buffer B in the Eppendorf tube and centrifuged at 20,000 × *g* for 5 min at 4 °C twice to purify the LD fraction. For HK-2 cells, cells were washed with ice-cold PBS before collection, followed by centrifugation at 1000 × *g* for 10 min at 4 °C. The samples were then suspended in buffer A plus 0.2 mM PMSF and incubated on ice for 20 min, followed by ultrasonic disruption. After centrifugation at 3000 × *g* for 10 min at 4 °C, and the PNS fraction and buffer B were transferred into a SW40 tube to centrifuge at 182,000 × *g* for 60 min at 4 °C. The LD fraction was carefully collected from the top band of the gradient and sequentially washed and purified three times by buffer B as mentioned above. Finally, an equal amount of the LD protein was analyzed by WB analysis. (4) *Confocal microscopy*: Confocal microscopy was carried out to detect the co-localization of LDs (BODIPY 493/503 neutral lipid stains), autophagosomes (MDC stains) and lysosomes (Lyso-tracker stains) the co-localization of RAB7 and LC3B and the co-localization of BECN1 and LDs (BODIPY 493/503 neutral lipid stains) in HK-2 cells. The analysis was performed using LSM 510 software (Zeiss).

### Cell IF

Treated HK-2 cells were fixed with 4% PFA for 5 min, permeabilized with ice-cold methanol for 10 min at −20 °C, blocked with blocking buffer (PBS + 1%BSA + 0.3% TritonX-100) for 1 h at 22 °C, and then incubated with primary antibodies against RAB7 (1:2000, Abcam, ab50533), LC3B (1:100, Proteintech, 18725-1-AP) and Beclin1 (1:100, Proteintech, 11306-1-AP) for 2 h at 22 °C. After washing with PBS, Alexa Fluor® 594-conjugated goat anti-rabbit or Alexa Fluor® 488-conjugated goat anti-mouse antibodies were applied for 1 h at 37 °C, followed by counterstaining with Hoechst to delineate the nuclei. Cells were then examined by LSM 780 META laser scanning microscopy.

### Extraction of nuclear protein

Separation of nuclear protein from the cytoplasmic protein was carried out using the Nuclear and Cytoplasmic Protein Extraction Kit (Beyotime) according to the manufacturer’s instructions. Briefly, HK-2 cells were resuspended in ice-cold hypotonic lysis buffer after intervention, and subcellular protein fractions extracted by homogenizing and centrifuging. Finally, an equal amount of the nuclear protein was analyzed by WB analysis. Primary antibodies specific for TFEB (1:1000, Proteintech, 13372-1-AP) were used. The bands were evaluated using a Tanon 5200 Multi instrument (Tanon Instruments, China). The band densities of target proteins were compared with those of Lamin B (1:1000, Proteintech, 66095-1-Ig) by using ImageJ.

### Bioinformatics and Nephroseq analysis

Normal human renal RNAseq data were obtained from Genotype-Tissue Expression (GTEx) for *AdipoR1* gene coexpression analysis, genes correlated with Pearson *r* > 0.6 and *p* < 0.05 were subjected to GO analysis via DAVID online tools (Version 6.8). Data for the analysis of the correlation between the gene expression of *AdipoR1* and renal function index (eGFR and Scr) were obtained by the publicly available data sets from the Nephroseq database (https://www.nephroseq.org/resource/login.html, V5).

### Statistical analysis

All statistical analysis was performed using the SPSS version 17.0 (SPSS Inc., Chicago, IL, USA) and GraphPad Prism 7.0 (GraphPad Software Inc., San Diego, CA, USA). The experimental data are expressed as the mean ± standard deviation. Differences between two groups were tested using *t-*test or Mann–Whitney *U*-test. For more than two groups, one-way analysis of variance (ANOVA) with Tukey’s post hoc analysis was used. The correlation between two variables was tested by Pearson’s correlation analysis. *p* < 0.05 was considered statistically significant.

## Data Availability

All data generated or analyzed during this study are included in this published article.
